# Nuclei on the Rise: When Nuclei-Based Methods Meet Next-Generation Sequencing

**DOI:** 10.3390/cells12071051

**Published:** 2023-03-30

**Authors:** Tamer Butto, Kanak Mungikar, Peter Baumann, Jennifer Winter, Beat Lutz, Susanne Gerber

**Affiliations:** 1 Institute for Pharmaceutical and Biomedical Sciences, Johannes Gutenberg-University, 55128 Mainz, Germany; 2 Institute of Human Genetics, University Medical Center Mainz, 55131 Mainz, Germany; 3 Faculty of Biology, Johannes Gutenberg-University, 55128 Mainz, Germany; 4 Institute of Molecular Biology (IMB), 55128 Mainz, Germany; 5 Leibniz Institute for Resilience Research (LIR), 55122 Mainz, Germany; 6 Institute of Physiological Chemistry, University Medical Center Mainz, 55128 Mainz, Germany

**Keywords:** nuclei isolation, next-generation sequencing, cell-type-specific isolation, epigenetics, transcriptomics, single-cell sequencing, single-nucleus sequencing

## Abstract

In the last decade, we have witnessed an upsurge in nuclei-based studies, particularly coupled with next-generation sequencing. Such studies aim at understanding the molecular states that exist in heterogeneous cell populations by applying increasingly more affordable sequencing approaches, in addition to optimized methodologies developed to isolate and select nuclei. Although these powerful new methods promise unprecedented insights, it is important to understand and critically consider the associated challenges. Here, we provide a comprehensive overview of the rise of nuclei-based studies and elaborate on their advantages and disadvantages, with a specific focus on their utility for transcriptomic sequencing analyses. Improved designs and appropriate use of the various experimental strategies will result in acquiring biologically accurate and meaningful information.

## 1. Introduction

The nucleus is the largest organelle in the eukaryotic cell, containing the genetic information (i.e., DNA) of a given organism [1]. In terms of its composition, the nucleus has its own double membrane nuclear envelope, which encapsulates the DNA-histone/DNA binding protein complexes (i.e., chromatin) in a well-organized state [2]. Within this state, the DNA forms a complex and high-order structure that ensures the efficient packing of the DNA in the nucleus and has a major effect on gene regulation, ultimately determining the given epigenetic and transcriptional profile of the cell. Due to the inherent properties and the content of the cellular nucleus, the number of studies that utilize nuclei for molecular investigations of cell-type-specific features has increased alongside the major research fields in molecular biology, such as genomics, transcriptomics and epigenomics. In fact, nuclei-based studies have come a long way since their initial discovery, exploration, isolation, and use as tools for molecular investigations [3,4,5].

In the last two decades, we have witnessed an upsurge in nuclei-based studies, particularly combined with high-throughput sequencing approaches. These studies aim to utilize isolated nuclei to identify both nucleus-associated properties and cell-type-specific features combined with supplementary techniques in molecular biology. The constant growth of next-generation sequencing (NGS), together with the steady reduction in sequencing cost, made the combination with nuclei-based studies both an affordable and increasingly powerful research tool [6]. Beyond this, the introduction of single-cell, as well as single-nucleus, sequencing technologies directed scientists to uncover the unique and heterogeneous molecular properties of a given cell population [7,8]. The rise of these research fields and technologies led scientists to focus on and develop novel tools to isolate cell-type-specific populations, focusing mainly on nuclei as tools for molecular investigations. However, with the unique features that nuclei-based studies offer, it is crucial to better understand the challenges and limitations of studies along this avenue. In the following, we will provide an overview of nuclei-based studies, highlighting the main high-throughput sequencing methodologies coupled with such studies, the reasons for their rise, and the upcoming challenges in the associated fields.

## 2. Nuclei-Isolation Procedures: From Cellular Dissociation to Nuclei Quality Check

Conceptually, the nucleus is a constituent of the cell, and therefore, its separation from the other cellular components will reduce the complexity of the biological system. Such a reduction in complexity can be used for two types of studies: 1. Investigation of nucleus-specific properties such as external and internal structural characteristics and/or 2. Use of the nuclei for downstream transcriptomic and epigenetic analyses as compared to the whole cell. Regardless of the type of investigation, high-quality, intact nuclei that is clean of debris should be obtained, and therefore the nuclei-isolation method is crucial. Below, we will emphasize key factors involved in the process of nuclei isolation, encompassing aspects such as cellular dissociation methodologies, commonly employed reagents, methodologies for isolating nuclei, and nuclei quality. All these factors will be contextualized with respect to the use of nuclei in subsequent genome-wide applications, with an emphasis on the interplay between nuclei-isolation methodologies and specific next-generation sequencing (NGS) approaches.

### 2.1. Cellular Dissociation Methods

Nuclei isolation from tissues or cultured cells requires the dissociation of cells into single-cell suspensions [9,10]. Various cellular dissociation methods have been developed to isolate nuclei from different sources, such as enzymatic digestion, mechanical techniques, and combination approaches.

#### 2.1.1. Enzymatic Digestion

**Figure 1:**
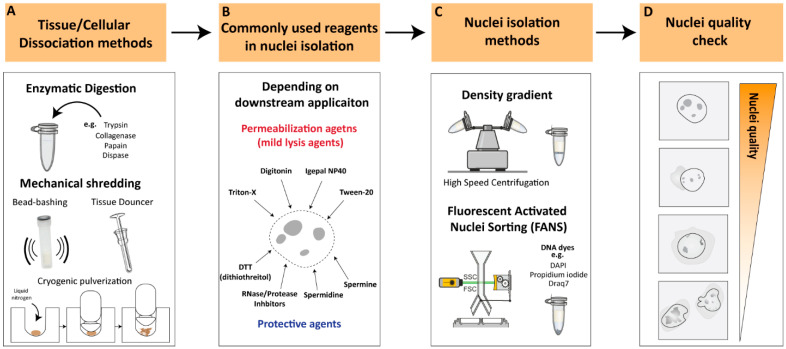
Key considerations for nuclei-isolation procedures (**A**). Tissue/cellular dissociation methods. Various cellular dissociation methods have been developed to isolate nuclei from different sources, such as enzymatic digestion, mechanical techniques and a combinatorial approach. (**B**). Reagents for nuclear permeabilization. Use of appropriate permeabilization buffers is crucial to ensure efficient and specific permeabilization of nuclei depending on downstream applications. (**C**). Nuclei-isolation methods. Density gradient centrifugations or flow-cytometry-based sorting are the most commonly used approaches for nuclei isolation. (**D**). Nuclei quality check. Illustration of high-quality nuclei are characterized by intact, round-shaped nuclei while low-quality nuclei often appear as small or fragmented particles with cytoplasmic debris or cellular aggregates. Low-quality nuclei could result in leakage of nuclear material from damaged or ruptured nuclei leading to contradictory observations.

Enzymatic digestion is one of the most commonly used methods for cellular dissociation. Enzymes such as trypsin, collagenase, papain and dispase can be used to break down cell–cell and cell–matrix interactions, releasing single cells [11,12,13,14] (Figure 1A). The strength of the enzyme needed for dissociation can vary depending on the type of tissue being dissociated. For instance, collagenase is commonly used for the dissociation of connective tissue, while trypsin is often used for the dissociation of epithelial cells [15]. Papain is another enzyme that is used for the dissociation of soft tissues [16]. Tissues that are difficult to dissociate, such as cartilage and bone, may necessitate stronger enzymes/reagents or suitable optimized protocols to achieve proper tissue dissociation and/or nuclear membrane permeabilization [16]. However, excessive exposure to enzymes can affect the viability and function of the cells, as well as the structure of nuclear components, which can affect downstream applications [17,18,19,20]. For instance, Denisenko and colleagues (2020) provided a systematic comparison of tissue dissociation protocols using both enzymatic digestions as well as nuclei-isolation approaches on adult mice kidneys, followed by single-cell and single-nuclei RNA-seq analyses [20]. The authors point out that enzymatic dissociation led to transcriptional changes consistent with a stress response, which was not observed with the non-enzymatic dissociation protocol. These observations are in agreement with previous studies reporting that enzymatic treatments could lead to major changes in cell-cycle status, induction of apoptosis, and structural alterations [18]. All these factors contribute to transcriptional changes that could lead to erroneous conclusions regarding the biological process under investigation [19]. Thus, it is important to optimize factors such as enzyme concentration, incubation time and temperatures to achieve efficient dissociation while maintaining the integrity of the nuclei.

#### 2.1.2. Mechanical Dissociation

Additional commonly utilized methodologies for cellular dissociation involve mechanical dissociation and are often employed in combination with appropriate reagents to ensure proper tissue dissociation. Mechanical dissociation techniques such as Dounce homogenization, ‘bead-bashing’ and cryogenic pulverization are often used with nuclei-isolation methodologies (Figure 1A). For instance, Dounce homogenization involves grinding tissue against a tight-fitting pestle, and is commonly used in nuclei-isolation procedures, particularly from softer tissues such as brain [21]. Bead-bashing involves the mechanical agitation of cells using glass or plastic beads, which can physically break apart the cells and release nuclei. Bead-bashing is not typically utilized in conjunction with nuclear isolation methodologies, but it has demonstrated efficacy in dissociating various tissues, contingent upon the intensity of mechanical force applied and sample properties [22,23]. Cryogenic pulverization involves freezing tissue in liquid nitrogen and then pulverizing it into a fine powder and can be used for a wide range of tissues, including hard-to-dissociate tissues [24,25,26,27]. However, it should be noted that mechanical dissociation techniques can also damage nuclei and may require additional steps to optimize nuclear isolation and quality. Overall, the choice of cellular dissociation method depends on the source of the cells or tissues, the specific application, and the sensitivity of the nuclear components to the dissociation method. The careful optimization of dissociation protocols is crucial to ensure the efficient and specific isolation of nuclei while preserving the integrity of the nuclear components.

### 2.2. Nuclear Permeabilization and Protective Reagents

Nuclei permeabilization is a critical step in many nuclei-isolation procedures that involve the isolation and analysis of nuclear components. To permeabilize nuclei, various buffers or reagents are used to break down the nuclear membrane and allow access to nuclear contents. Commonly used buffers for nuclei permeabilization include mild lysis agents such as Triton X-100, Tween-20, digitonin and Igepal NP40 [27,28,29,30] (Figure 1B). These non-ionic detergents exhibit distinct chemical structures and properties that can solubilize the lipid bilayer of the nuclear membrane, allowing other molecules to enter the nucleus without disrupting the structure of the nuclear components. Often these detergents are used in combination with other detergents in relatively low concentrations (<0.5%) to improve their solubilizing properties, depending on the downstream application [28]. The selection of an optimal detergent is influenced by factors such as the source of the sample, input size, and downstream applications. The present review will provide an overview of multiple investigations utilizing nuclei-isolation protocols, thereby allowing the reader to identify the most appropriate methodology for their specific research requirements.

To ensure efficient and high-quality nuclei without causing damage to nuclear components, it is recommended to supplement buffers with protective agents against nuclear degradation, depending on downstream applications (Figure 1B). Spermidine and spermine, which have antioxidant and stabilization functions on the chromatin structure, are often used as protective agents in nuclear isolation [27,31]. Another commonly employed protective reagent is DTT (dithiothreitol), a reducing agent utilized in nuclei-isolation protocols to preserve the integrity of the chromatin structure and promote the isolation of intact nuclei [17,20,27,30]. Additionally, RNAse and/or protease inhibitors are commonly added to the working buffer, depending on the downstream application, to prevent RNA and/or protein degradation during nuclear isolation processing. These reagents facilitate the isolation of intact nuclei, leading to the improved quality and yield of RNA samples for downstream analysis.

### 2.3. Nuclei-Isolation Methodologies

Nuclei-isolation procedures were established over half a century ago and involve density gradient centrifugation to visibly separate the nuclei from the rest of the raptured cell components [32,33,34]. Density gradient centrifugations rely on decreasing density solutions that amass the migrating target components according to their density during the centrifugation process [35]. The enveloped nuclei, containing the tightly packed DNA/proteins (i.e., highly dense structures), can be separated from the raptured and less-dense cytoplasmic compartment [36]. Density gradient nuclei-isolation approaches involve high-speed centrifugation, which results in a relatively intact, debris-free nuclei population and, therefore, is ideal as a rapid nuclei-isolation procedure (Figure 1C). However, nuclear integrity can greatly differ depending on the isolation method, nuclei storage buffer, and suspension time in intermediate buffer [37]. In recent years, an increasing number of studies have reported optimized nuclei-isolation procedures, which vary depending on the type of organism, tissue, or cell from which the nuclei were isolated (See ‘Critical considerations for efficient nuclei isolation from diverse biological sources’).

An additional commonly used methodology for nuclei isolation is flow-cytometry-based nuclei sorting [37,38]. The principle of flow cytometry is based on the ability of cells or nuclei to pass through a stream of fluid and be sorted according to size, granularity, and fluorescence properties (Figure 1C). In the case of nuclei isolation, flow cytometry can be used to sort and isolate nuclei based on their DNA content, which is typically measured by staining with fluorescent DNA dyes such as propidium iodide, Draq7 or 4′,6-diamidino-2-phenylindole (DAPI) [39,40]. Fluorescent DNA stains are often used in combination with FANS (Fluorescence-Activated Nuclei Sorting) to isolate nuclei based on their fluorescence signal and size, allowing for the separation of intact nuclei from other cellular components and debris [37]. FANS can be used to isolate nuclei from various sources, including tissues and cultured cells, and it has several advantages over other methods, such as the ability to sort nuclei based on their DNA content and the ability to sort multiple populations of nuclei simultaneously [21]. However, flow cytometry also has some limitations, such as the need for a specialized instrument, the requirement for a large number of cells/nuclei, and the potential for nuclei damage due to shear forces generated during the sorting process.

Overall, flow cytometry is a useful and powerful tool for nuclei isolation that can be used in conjunction with downstream applications such as genomics, transcriptomics, and epigenomics.

### 2.4. Nuclei Quality Check

Nuclei quality checking is a critical stage for evaluating and ensuring the integrity and purity of nuclei prior to their use in downstream applications, such as single-cell or genome-wide sequencing assays. The quality check of isolated nuclei can be conducted during or after the isolation procedure by using several methods. One way to assess nuclei quality is by using fluorescent dyes that specifically label the DNA or RNA of the nuclei, such as DAPI, and assess the nuclear integrity through microscopic examination or flow cytometry analysis, where the nuclei are identified based on their size and DNA content. High-quality nuclei can be characterized by intact, round-shaped nuclei with minimal damage or debris from other cellular components (Figure 1D). On the other hand, low-quality nuclei often appear as small or fragmented particles under the microscope and may contain cytoplasmic debris or cellular aggregates. Low-quality nuclei could result in the leakage of nuclear material from damaged or ruptured nuclei, leading to the contamination of cytoplasmic RNA, proteins, and other cellular components, potentially confounding downstream applications (Figure 1D). Thus, it is essential to perform quality control measures to ensure that the isolated nuclei are of high quality before proceeding with downstream applications.

### 2.5. Critical Considerations for Efficient Nuclei Isolation from Diverse Biological Sources

#### 2.5.1. Nuclei Isolation from Distinct Organisms

With the increased use of genome-wide sequencing technologies, the interest in the isolation of single, separated nuclei has increased accordingly. Such an interest is extended to distinct organisms containing dissimilar genetic compositions. Early nuclei-isolation procedures, which involved density gradient centrifugations, focused mostly on the isolation of nuclei from mammalian tissues [33,35,41]. In fact, to this day most of the current studies that report optimized nuclei-isolation procedures are associated with mice or human dissected tissues [21,27,29,32,42]. However, an increasing number of studies report alternative nuclei isolation from distinct organisms, including invertebrate model organisms such as *Drosophila melanogaster* [43], *Caenorhabditis elegans* [44,45], non-model organisms such as diatoms [46] and a variety of plant species [47,48,49].

**Figure 2:**
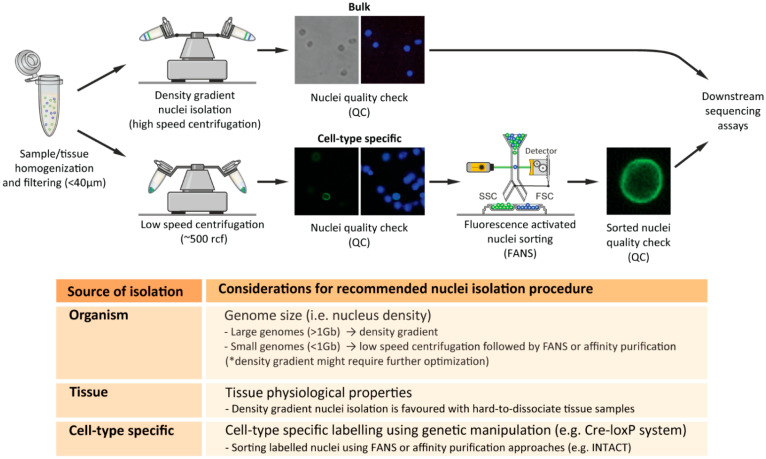
Overview of nuclei-isolation strategies and consideration of isolation method according to source material.

Interestingly, we observed an increased association of high-speed density gradient nuclei-isolation procedures with mammalian tissues as well as distinct plant species. On the other hand, the association of high-speed density gradient isolation was not clear in nuclei-isolation procedures from invertebrates or non-model organisms where low-speed centrifugation procedures, coupled with sucrose or alternative density gradient solutions (e.g., Optiprep), were favored [43,44]. Moreover, optimized nuclei-isolation procedures are shifting towards non-model organisms, emphasizing the necessity of specialized organism-specific nuclei-isolation procedures. For instance, a recent study reported a nuclei-isolation method from unicellular eukaryotic microalgae (i.e., diatoms), where the authors remarked that ‘Density gradient centrifugation methodologies were tested to separate nuclei from the other components…but none of the attempts allowed an efficient separation of nuclei from cell debris (and bacteria, when present)’ [46]. One of the potential reasons for unsuccessful density gradient isolation could be the altered nuclei densities due to varying genome sizes between distinct organisms. Mammalian species often comprise large genomes (>1 Gbp), and therefore the dense nuclei can be separated easily with appropriate density gradient protocols. Invertebrates often comprise smaller genomes (<1 Gbp), and therefore density gradient procedures should be optimized according to the reduced density of the nuclei (Figure 2). On the other hand, plant nuclei also contain relatively small genomes (depending on the plant species); however, previous studies using density gradient procedures for plant nuclei isolation use adjusted gradients, enabling the efficient isolation of nuclei [47]. Taken together, developing organism-specific nuclei-isolation procedures is essential for the rapid and efficient recovery of high-quality intact nuclei.

#### 2.5.2. Nuclei Isolation from Distinct Tissues

Within an organism, distinct tissues comprise distinct cellular and physiological properties, which make their cellular isolation and processing challenging. For these reasons, the selection of nuclei versus whole-cell isolation procedures can vary according to the selected tissues. Previous studies addressed the potential rationale for the selection of isolation method according to the source of material, arguing that in contrary to nuclei-isolation procedures, whole-cell separation and isolation of single units often require enzymatic or substance treatments [9,10]. Remarkably, the understanding of nuclei versus whole-cell comparisons, including material isolation methods and transcriptional analyses, has been obtained recently from single-cell/nucleus comparison studies [50,51,52]. (Further information will follow later in this review under the section ‘Single-cell and single-nucleus sequencing studies’). Furthermore, single-cell/-nucleus comparison studies stated that nuclei-isolation procedures are advantageous for certain hard-to-dissociate tissue types such as brain, bone, or adipose tissues [51,52] (Figure 2). This is probably due to the properties of the tissues where enzymatic dissociation would be inefficient, but the isolation of nuclei, which includes removing the external structural layers of the cell, would instead be very efficient. Therefore, tissue type will often dictate the selection of the isolation method, according to the experimental design and biological question investigated.

#### 2.5.3. Cell-Type-Specific Nuclei Isolation

A key challenge in biology is to understand the origin and maintenance of cellular diversity within a single organism, despite the identical genetic composition. The interest in such phenomena led scientists to explore the molecular attributes of specific cell populations in order to identify their characteristics and varying roles within an organism. To further comprehend such cellular heterogeneity, several methodologies have been developed to isolate cell-type-specific populations based on their unique molecular features (e.g., specific gene and/or protein expression), thereby uncovering their unique genome-wide characteristics within a seemingly similar cell population. The use of nuclei has been demonstrated as a valuable tool, particularly when combined with genetic manipulation (e.g., Cre-loxP system) for cell-type-specific labelling [53]. For instance, the technique ‘Isolation of Nuclei in Tagged Cell Types’ (INTACT) is an affinity purification-based method that isolates genetically defined populations that express a fluorescently tagged nuclear membrane protein (e.g., Sun1GFP) [54,55] (Figure 2). While INTACT was initially developed in plants, its use has been adapted for other model organisms and has gradually evolved towards the analysis of multiple cell types within the mammalian system, primarily within the intensively investigated mammalian brain [37,43,56,57,58]. Another frequently used approach for cell-type-specific nuclei isolation is using flow-cytometry-based procedures using endogenous or exogenous fluorescence labeling approaches [59]. For instance, one frequently used method for neuronal isolation is using the neuronal nuclear marker NeuN [60], which has been used for a long time. It was not until 2012 that the terminology FANS was coined, emphasizing the requirement of nuclei-specific sorting procedures [61]. A detailed summary of the methodologies used to isolate cell-type-specific populations, including nuclei, and their association with genome-wide transcriptomic and epigenomic analyses is reviewed in [59]. Overall, nuclei-based approaches have entered the realms of day-to-day experimental routine, yet so far, with relatively little information about their distinct properties and differences compared to whole-cell studies. In the following, we will highlight the main high-throughput sequencing methodologies coupled with nuclei-based investigations, the potential reasons for their popularity, and upcoming challenges.

## 3. Association of Nuclei with Next-Generation Sequencing

### 3.1. Genomics and Epigenomics

It has become clear that the nuclear content has a major role in cellular function and regulation. The more frequent utilization of nuclei has gone hand-in-hand with an elevated interest in genomics and epigenetics, as well as significant technological improvements in various high-throughput NGS platforms [62].

Such technologies have allowed for the detection of molecular changes on a genome-wide level, providing both comprehensive databases of specific cell identities as well as locus-specific alterations within a given experimental condition. The curiosity to uncover the internal function and content within the nuclei of distinct cellular populations led to the growth of the field of epigenetics and the development of experimental techniques dedicated to uncovering the multifaceted mechanisms of gene regulation. For instance, the mechanisms of DNA methylation and post-translational modification (PTMs) of histones rely on the deposition of chemical modifications on the DNA or DNA-associated proteins (e.g., histones) within the nucleus [63]. These mechanisms control gene expression, which influences cell function, and ultimately give each cell type its unique epigenetic and transcriptomic identity. The isolation of nuclei has been shown to be sufficient for conducting a variety of epigenetic assays as many of these assays require access to the contents within the nuclei. For example, techniques such as ChIP-seq [64], Hi-C [65], and ATAC-seq [66] are designed to investigate the dynamic chromatin structure and chromatin-bound proteins that reside within the nucleus. Regardless of the specific downstream application of these assays, isolated nuclei have been demonstrated to be a suitable starting material for various epigenetic analyses. Given the growing interest in epigenetic regulatory mechanisms, particularly in the context of high-throughput sequencing, the use of nuclei has become an attractive option for these applications.

### 3.2. Transcriptomics

#### 3.2.1. RNA-seq

The process of transcription is the first stage that transforms biological information (i.e., genetic code) into a translated outcome or product (i.e., protein). The field of transcriptomics aims to monitor and quantify the complete set of transcripts, including coding and non-coding RNAs, within a given cell at a given condition [67]. The investigation of the transcriptome is crucial for understanding the functional elements of the genome and their roles within a cell or tissue. RNA-seq enables the assessment of genome-wide transcriptional states and hence has become the primary methodology to investigate the transcriptome and all its variations. Since the advancement of the next-generation sequencing approaches, we have seen a dominant use of the RNA-seq, which keeps increasing over time.

#### 3.2.2. Nuclear RNA-seq

On the other hand, nuclear RNA-seq (nucRNA-seq), which refers to the sequencing of RNA isolated from the cell nucleus, has not been used nearly as frequently, which might reflect the novelty of such a sequencing technique. If we contemplate the association between nuclear RNA and next-generation sequencing, the first evident description of nucRNA-seq was reported by Mitchel and colleagues (2012), who took the opportunity to compare the nuclear transcriptome of erythroid cells with RNA polymerase II (RNAPII) occupancy [68]. Already at that time, the authors reported a large fraction of unspliced transcripts, which was detected by nucRNA-seq, hence foreseeing some of the challenges that will be discussed below.

#### 3.2.3. Association of nucRNA-seq in ‘Multi-Omics’ Studies

An increasing number of studies seek to combine multiple ‘omics’ approaches within a given experimental design to identify specific and shared molecular properties of a given cellular population [69,70]. A frequent example of such experiments is the association of RNA-seq with epigenetic methodologies such as chromatin accessibility or DNA methylation within a single experimental setup. Similarly, the application of nucRNA-seq has been applied as a ‘multi-omic’ approach. For instance, Chongtham and colleagues (2021) performed a literature review comparing the use of two nuclei sorting techniques, INTACT and FANS, including subsequent molecular analyses used in conjugation with the investigated techniques [37]. The authors observed that ~60% of the analyzed studies combined more than one sequencing technique. This illustrates the frequent association of various ‘omics’ approaches within the same study, particularly in nuclei-based research. Using these associations, scientists can obtain broader and more comprehensive insights into particular cellular mechanisms per given condition. Depending on the biological question investigated and distinct sample requirements, nuclei are often coupled with additional approaches such as FANS or INTACT to isolate specific populations and diversify their use in various sequencing methodologies. Overall, the rise of the mentioned sequencing techniques and their association with nuclei is undoubtedly a landmark in the fields of molecular genomics, transcriptomics and epigenomics, which have already shifted towards single-cell and, also, increasingly, single-nucleus sequencing.

### 3.3. Single-Cell and Single-Nucleus Sequencing Studies

Single-cell studies have taken on an essential role in biological research, providing unprecedented insights into the characteristics of individual cells within a population. One of the most frequently utilized approaches is single-cell RNA-seq (scRNA-seq), which reveals the transcriptome of individual cells and highlights the heterogeneity among seemingly identical cell populations. However, in parallel, studies utilizing single-nucleus RNA-seq (snRNA-seq) have gradually risen in number as well, and with them, the inevitable question: what are the differences between scRNA-seq and snRNA-seq?

The first application of snRNA-seq occurred one decade ago, providing the basis for further developments of snRNA-seq methodologies [71]. Grindberg and colleagues (2013) applied the first snRNA-seq to uncover the dynamic transcriptome of mouse neuronal progenitor cells [71]. The authors noted that single-nuclei sequencing provides a unique insight into the exploration of neuronal transcriptomes since it ‘avoids requiring isolation of single-cell suspensions, eliminating potential changes in gene expression due to enzymatic-cell dissociation methods’. In their analysis, the authors took the opportunity to compare bulk nuclei versus bulk cells as well as single nucleus versus single cell, opening the avenue to the exploration of nuclear transcriptomics at the single-cell level.

Several studies followed and investigated the comparison between single nuclei and single cells, providing extensive information about the experimental design of such experiments, including material collection/isolation (of cells and nuclei) as well as a comparison of downstream analytical tools and biological differences between these two compartments. Comparing single-nucleus and single-cell RNA-seq within a single experimental scheme has become frequent [51,52,72,73,74,75]. The fundamental differences in RNA composition between nuclei and whole cells are noteworthy, which inevitably led to studies that explore and uncover such differences in various biological systems. For instance, Lake and colleagues (2017) compared single-nucleus RNA sequencing (snRNA-seq) and single-cell RNA sequencing (scRNA-seq) to investigate differences in gene expression between individual nuclei and intact cells in the human brain. They found that snRNA-seq was able to detect unique features of gene expression that were not detected by scRNA-seq. In particular, snRNA-seq was more sensitive to detecting low abundance transcripts and transcripts that were restricted to certain cell types. However, snRNA-seq also showed a lower overall detection rate of transcripts compared to scRNA-seq [72]. Bakken and colleagues (2018) compared scRNA-seq and snRNA-seq from the mouse visual cortex [74]. The authors suggest that although the number of transcripts detected from scRNA-seq is higher than snRNA-seq, the latter can be similarly associated with neuronal cell types. Interestingly, the authors highlighted that the incorporation of introns was required for comparable clustering analysis between snRNA-seq and scRNA-seq. They speculated that this is due to the long genes known to be brain-specific and that this helps defining the neuronal population analyzed [74]. In another study, Slyper and colleagues (2020) provided a comprehensive overview covering various cancer cell types, distinct protocol strategies, tissue acquisition, and sequencing methodologies of scRNA-seq and snRNA-seq [51]. In this study, the authors remark about the choice of using either single-cell or single-nucleus RNA-seq, stating, ‘The choice between scRNA-Seq and snRNA-Seq is typically driven by sample availability, logistics, and biological question’. The advantages of snRNA-seq include the decoupling of sample acquirement and processing, high recovery from tissues difficult to be dissociated, and sample multiplexing within specific approaches such as Drop-seq [51,76,77]. Nevertheless, the authors highlighted the importance of testing several tissue-specific dissociation methods as the output can vary depending on the type of dissected tissue and the processing procedure. In a similar study, Ding and colleagues (2020) provided a systematic comparison of single-cell and single-nucleus RNA-seq, focusing on the isolation method, sequencing platform, and computational analyses [52]. Comparable with previous studies, the authors remarked that the selection of single nuclei can be an important strategy that could be directed towards complex tissue types showing reduced gene expression alterations, as compared to cellular dissociation methods. Overall, these studies highlight the importance of considering the strengths and limitations of different single-cell analysis techniques when investigating gene expression patterns in complex tissues.

#### Lessons from Single-Cell/-Nucleus Sequencing Analyses

With the unprecedented data that single-cell/-nucleus sequencing provides, such technologies have shed light on the limitations that could originate during the sample processing step, as well as material-specific biases that arise according to the biological source (i.e., nuclei or whole cell). For instance, in a study that performed scRNA-seq on muscle stem cells, the authors reported alterations in gene expression in a subpopulation of cells that were caused by the sample dissociation procedure [78]. The subpopulation was characterized by a high-level expression of immediate early genes (IEGs) known to be activated following exposure to a stimulus, shown to be inflicted by the dissociation protocol [77]. One can only assume that such changes in IEGs could also occur in highly heterogeneous cell populations, such as glial cells and/or neurons, which likewise are vastly influenced by external stimulations [79]. In another example, a study that aimed to compare transcriptional microglial activation signatures using single-cell and single-RNA-seq revealed the inadequacy of snRNA-seq for detecting such activation signatures, which were depleted in nuclei as compared to scRNA-seq [80]. Such an observation, consistent with previous studies [81,82,83], demonstrates the inherent technical limitation of nuclei in association with specific cell populations. These limitations can only add up to the already existing challenges that single-cell data comprise, and therefore, the consideration of the biological constituent (nuclei/cells) should be reflected as well for meaningful and accurate biological research [84]. Notably, all the considerations stated in the single nucleus/cells section can be applied to bulk analyses as well, especially when considering transcriptome analyses. It is most likely that the combination of various factors described here ultimately leads to the selection of nuclei as simple yet effective biological components for the investigation of epigenetic and transcriptomic states within cell-type-specific populations. Comparing the transcriptomes of single nuclei and single cells can provide a great source of information to better understand their transcriptional differences and design more precise transcriptomic analyses. However, at the transcriptomic level, there are distinct potential challenges that we believe should be addressed further. For a summarized overview specific to the use and limitations of single-nucleus RNA-seq, see [85].

## 4. Limitations of Nuclei-Based Studies

Unlike epigenetic studies, the data analysis of nuclear transcriptomics poses specific challenges as nuclear RNA has distinct properties compared to total or cytoplasmic RNA. As illustrated previously, several studies have compared the differences between nuclear and cytoplasmic RNA fractions [86,87,88]. Depending on the subject of investigation, such differences may be highly significant and thus require careful consideration prior to sequencing. Comparing nuclear and cytoplasmic RNA fractions is vital to comprehend, design, and utilize better high-throughput sequencing approaches, as each has its advantages and biases. The following section summarizes the major challenges to be considered when comparing nuclear and whole-cell or cytoplasmic RNA, highlighting the different properties and the major implications of each fraction for RNA-seq analysis.

### 4.1. RNA Content-RNA Population Bias between Nuclei and Whole Cell

Within the eukaryotic cell, transcription is often coupled with RNA post-transcriptional processing steps and transportation to the cytoplasm [5]. In the specific case of mRNAs and many lncRNAs, the RNA splicing machinery removes intronic regions and joins exonic segments, frequently involving the use of alternative splice sites and generating a host of alternative transcripts [89]. Already at this stage, the composition of the various RNA populations is different between the nuclear and cytoplasmic fractions since the transcripts contain a profoundly different ratio of exonic and intronic sequences, with only a minority of intronic sequences finding their way into the cytoplasm [90]. Generally, nuclear and cytoplasmic fractions contain overlapping RNA transcripts; however, there are also many transcripts that are strongly enriched in one fraction. For instance, nuclear mRNA retention serves the accumulation of specific transcripts in the nucleus, where they may be sequestered to prevent translation [91]. In general, however, mRNAs were found to be more equally distributed between the fractions, whereas lncRNAs, snoRNAs, and snRNAs were more abundant in the nuclear fraction [92,93]. If we consider recent studies that utilized nuclear RNA-seq, we notice that such RNA populations predominantly feature in the transcriptomic analyses [37,94]. This suggests that the transcriptional analyses of nuclei could be biased towards these nuclear RNA populations. Therefore, all sequencing approaches that deal solely with nuclear RNA should be critically analyzed. The study of other RNA classes, such as rRNA or small RNAs, and the analysis of RNA modifications, should also be handled with caution when nuclear RNA is analyzed. Future studies are needed to fully uncover the properties of nuclear and cytoplasmic (or whole-cell) RNA and their different populations and modifications.

### 4.2. Experimental Design of Nuclear RNA-seq-Sequencing Depth and Analysis of Exon Versus Intron Reads

If we consider the ratio of intronic to exonic sequences in nuclear RNA, the nuclei contain largely unspliced transcripts (i.e., containing introns), while the majority of spliced transcripts will be in the cytoplasm. In metazoans, this ratio is further skewed at the nucleotide level as introns are frequently thousands of nucleotides long, many times longer than exons [95,96]. Therefore, an important aspect to consider is the sequencing depth and read length since the distribution of reads will vary dramatically depending on the presence or absence of introns. Thus, it is crucial to select a suitable sequencing depth depending on the biological question investigated. Nuclear RNA-seq will require higher sequencing depth to better capture the diluted exonic fraction compared to conventional RNA-seq. For instance, in the first reported nuclear RNA-seq, the authors analyzed the percentage of intron and exon reads [68]. The authors reported that: ‘nucRNA-Seq library showed a strong bias toward intronic reads as introns are generally much larger than exons (36% exonic)’. In the study by Fernandez-Albert and colleagues (2019), the authors utilized up to 80 million reads for nuclear RNA-seq to obtain sufficient exonic reads for subsequent transcriptional analysis. Nevertheless, 80% of the aligned reads were intronic, illustrating the bias of nucRNA-seq towards unprocessed, primary transcripts [94]. Hence, the sequencing depth is an essential factor to be considered in the interpretation of nucRNA-seq data [97].

### 4.3. Nuclear RNA Quality and Library Preparation Strategies

Two important aspects to consider before sequencing are the assessment of RNA quality and the selection of an appropriate RNA library preparation strategy. Both constitute major potential challenges that may influence nucRNA-seq data.

#### 4.3.1. Nuclear RNA Quality

The RNA integrity number (RIN) has been widely used as an RNA quality measurement prior to library preparation and sequencing. RIN is an algorithm tool that measures the ratio of the area under the 18S and 28S rRNA peaks compared to the total area under the graph on a bioanalyzer instrument [98]. In such a way, RIN provides an indication of the RNA degradation state of specific samples. RIN values of >7 are often accepted as sufficient RNA quality for sequencing, whereas values below 7 indicate a level of degradation that may impact the quality of the sequencing results. RIN values will vary depending on the handling, storage conditions, and concentrations of the available material. As Krishnaswami and colleagues (2016) noted, the isolation of total bulk RNA, which is often isolated from high cell numbers, results in higher RIN values and hence higher-quality RNA. On the contrary, the authors remarked that the isolation from nuclei can result in varied RIN values [29]. We reason that those initial nuclei input materials will affect the concentrations of the RNA and, consequently, the RIN values as well. Conceptually, higher concentrations of RNA might result in a more constant ratio of 28S:18S rRNA peaks, which can be more accurately detected by the measurement device. In contrast, low input material will result in lower RNA concentrations that could largely affect the proper detection of 28S:18S rRNA peaks. These changes may be sufficient to result in varied RIN values that ultimately will prevent the subsequent sequencing and analysis of such samples. Previous studies demonstrated the effects of RNA degradation and differential RIN values of whole cells on the quality of conventional RNA-seq [99,100,101,102]. Overall, these studies conclude that useful information can still be obtained from highly degraded samples [100]. However, it is important to be aware of the potential biases that can arise during the experimental handling. It is recommended to minimize intergroup differences in RNA quality (i.e., in RIN values) to obtain comparable data. The emphasis of such studies should be directed towards critical data analysis, with an awareness of the potential effects of RNA quality on data interpretation [102]. Krishnaswami and colleagues (2016) go further and suggest ‘if RNA with a RIN score of <7 is all that is available, it should be tested, and it may still yield valuable data’. We agree with this view. However, we argue that further studies should determine the potential reasons for nuclear RNA variations concerning nuclear RNA degradation and their influence on nuclear RNA-seq.

An additional point to be addressed regarding nuclear RNA quality is the attention given to nuclear rRNA (ratio) using the bioanalyzer. One apparent question to investigate is whether the levels of nuclear 18S and 28S rRNA accurately correspond to the total rRNA content measured across the entire cell. Surprisingly, we observed little evidence for the quantification of the 18S and 28S rRNA in the nuclear fraction as compared to the cytoplasm or total cell. One report has shown that the levels of 18S and 28S rRNA in HeLa cells were found in higher amounts in the cytoplasmic fraction as compared to the nuclear fraction [103]. These results suggest that indeed the levels of nuclear rRNA might not be representative as measured in the whole cells. However, further studies should examine such an observation for a more detailed conclusion.

Another aspect that requires consideration is the technical detection of the rRNA peaks as provided by the bioanalyzer manufacturer. If we examine the application document ‘RNA integrity number (RIN)-Standardization of RNA quality control’ provided by the manufacturer (Agilent technologies), we did not encounter any statements regarding the use of nuclear RNA for quality control assessment [104]. In fact, it is stated that: ‘The RIN software algorithm allows for the classification of eukaryotic total RNA’ where the development of the RIN tool was established using ‘…Input data included approximately 1300 total RNA samples from various tissues…’. These statements suggest that nuclear RNA was not assessed with the available algorithm and therefore is not fully suitable for the quality control of nuclear RNA until fully examined. Nevertheless, we believe that many of the nuclei-based studies initially measure the RIN values as a routine RNA quality measure before library preparation and sequencing. However, due to variable RIN outcomes, which often do not reach the accepted value, such measurements are not always reported in recent studies. We noticed that several studies explicitly report the RIN values for nuclear RNA [29,57,73,105], while others do not provide such information [68,94,106,107,108,109]. These inconsistencies might reflect our partial understanding of RIN values together with nuclear RNA as compared to whole-cell RNA. Therefore, future studies should evaluate the appropriateness of RIN as a quality indicator for nuclear RNA. As previously noted, all RNA populations’ content can vary largely depending on the consideration of nuclear and whole-cell RNA content, including the rRNA fraction. For instance, further investigations should determine if the proportions of rRNA present in the nucleus and whole cell are comparable. It is possible that nuclear RNA does not reflect a constant ratio of rRNA to total RNA (depending on transcription speed, transcript processing, transport (to cytoplasm) speed or leakage from the nucleus to the cytoplasm), and therefore, nuclear RNA RIN values might not be representative of nuclear RNA quality.

#### 4.3.2. RNA Library Preparation Strategies

An additional aspect that should be thoroughly considered is the choice of RNA library preparation strategy for nuclear RNA material. Multiple commercially available RNA library kits provide different approaches for selecting specific RNA populations [110]. Here, we will focus on two main strategies for RNA library preparation: poly(A) enrichment and rRNA depletion strategies. Poly(A) enrichment strategies are most commonly used for RNA-seq, underlining the selection of poly-adenylated transcripts, such as protein-coding genes (mRNAs) and lncRNA [111]. This approach relies on high-quality (non-degraded) RNA to select polyadenylated transcripts since fragmentation or extremely long polyA regions can lead to 3′UTR coverage bias [112]. Using this strategy, other RNA populations such as non-polyadenylated RNA (e.g., miRNAs) will not be detected due to the absence of a polyA tail. As a substantial amount of splicing occurs co-transcriptionally and therefore prior to polyadenylation, poly(A) selection will affect which RNAs are enriched in their unspliced or spliced form. As the relative timing of intron removal and polyadenylation is subject to regulation, this may have profound implications for transcript quantification. In contrast, rRNA depletion strategies rely on the exclusion of rRNA by bead separation or selective degradation [92]. Although this approach requires high amounts of total RNA, commercial alternatives are available for low amounts of input material [113,114]. The choice between the different RNA library preparation strategies should be suitable for the investigation regardless of any selected type (nuclei or cells) or input amount (high or low).

## 5. Computational Analyses of Classical (Whole Cells) and Nuclear RNA-seq

**Figure 3:**
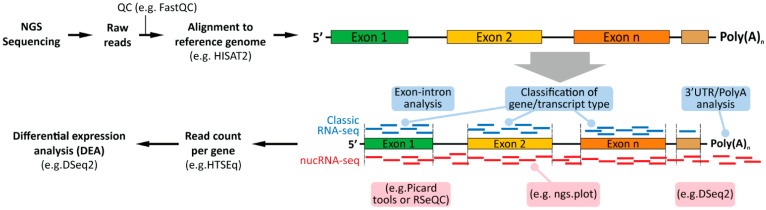
Overview of data analysis of classic and nuclear RNA-seq. Notably, all the mentioned tools can be applied both on nucRNA-seq and classic RNA-seq.

To our knowledge, there are no reports that describe the necessity of a specified pipeline for nuclear RNA-seq. However, specified analytical steps can be designated according to the requirements of nucRNA-seq (Figure 3). To start with, following sequencing, both classical RNA-seq and nucRNA-seq data analysis comprise of an initial quality check of the raw reads generated during sequencing. The quality check can be performed using FastQC followed by alignment to the selected reference genome/transcriptome, using a read alignment tool such as Bowtie [115], STAR aligner [116] or HISAT2 [117], to name a few. At this stage, an intron-integrated reference transcriptome can be utilized specifically for nucRNA-seq data to assess exon-intron coverage since nucRNA-seq reads consist of a higher fraction of intronic than exonic reads [90,118]. Following alignment, read counts (per gene/transcript) can be calculated using HTSeq [119], and distinct strategies can be applied depending on the material sequenced. For instance, nucRNA-seq data analysis could consist further of intronic-exonic mapping analysis using Picard tools ‘CollectRnaSeqMetrics’, QuasR ‘qCount’ or RSeQC ‘read_distribution.py’ utilities for intronic and exonic read mapping calculations. Additionally, as previously described, depending on the library preparation strategy, nuclear mRNA might contain longer 3′ UTR/polyadenylated fragments as compared to cytoplasmic mature mRNA, which might influence downstream analyses. Therefore, it is important to inspect the read occupancy profile and coverage across the expressed genes. For this, ‘ngs.plot’ can be utilized to visualize and assess the coverage biases at the transcriptional start and end sites (TSS and TES, respectively) as well as the coverage across the intron/exon regions [120]. Read normalization and differential expression analysis allow for the identification of distinct gene expression levels between distinct conditions. Tools such as DESeq2 [121], limma [122], or edgeR [123] can be used for such. Lastly, nucRNA-seq data could comprise a large number of small RNAs and long-noncoding RNAs, and therefore, it is important to monitor such differences to identify the biases present.

## 6. Discussion and Future Perspective

**Figure 4:**
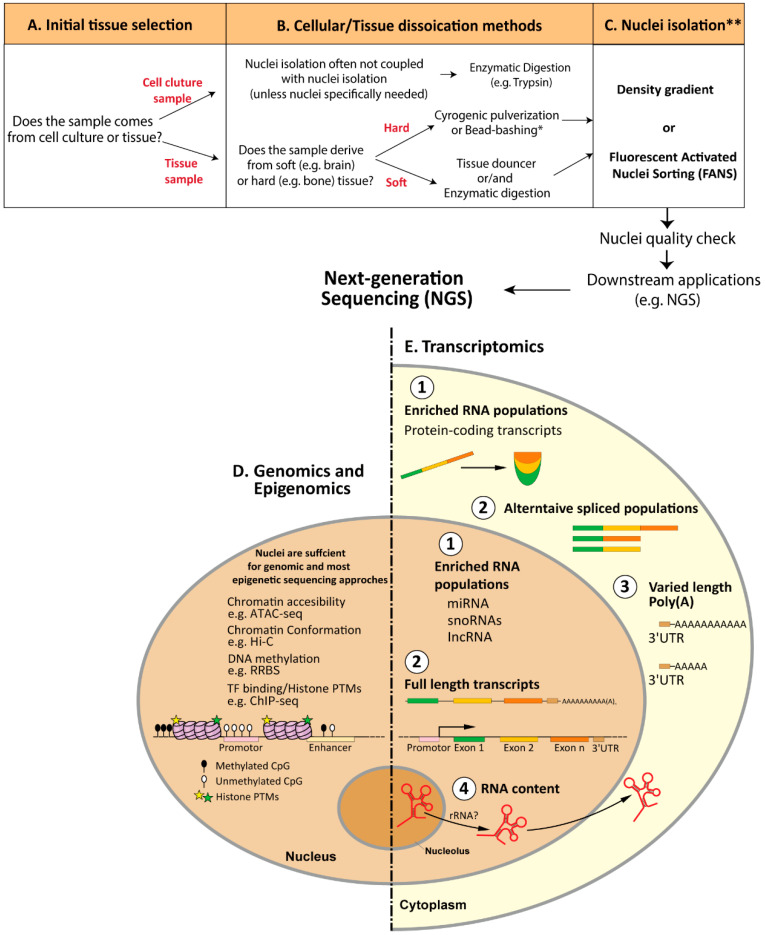
Overview of the application of nuclei-based studies with next-generation sequencing approaches. (**A**–**C**)**.** Guideline for selecting the optimal nuclei-isolation pipeline coupled with NGS. (**A**). Selection of sample material in vitro or in vivo. (**B**). Tissue/Cellular dissociation methods. According to the sample type, samples can be dissociated with mechanical dissociation (e.g., tissue samples) or enzymatic digestions (e.g., cell culture). Soft tissues (e.g., brain) can be homogenized with a tissue Douncer (in combination with enzymatic digestion if required) whereas hard tissues require stronger dissociation methods such as cryogenic pulverization or bead-bashing. * Dissociation of hard tissue types might require further optimization steps. (**C**). Nuclei-isolation methods. Density gradient and FANS are the most frequent methods to isolate nuclei. ** The selection of nuclei-isolation method depends on cell type, input material and downstream application. (**D**). Genomics and Epigenomics. The use of nuclei is sufficient for most epigenetic sequencing approaches as the content of interest (e.g., gDNA, histones and chromatin-associated modifications) is located within the nucleus. (**E**). Transcriptomics. Differences between nuclear and cytoplasmic transcripts regarding 1. Distinctly enriched RNA populations, 2. Distinct intronic and exonic transcript ratio, 3. Distinct length of poly-adenylated transcripts, 4. Distinct rRNA content.

In this review, we present a comprehensive analysis of various factors that must be considered while isolating nuclei. These factors include the selection of appropriate sample type (Figure 4A), cellular dissociation methodology (Figure 4B), and optimization of nuclei-isolation techniques (Figure 4C) depending on the sample type. Additionally, it is crucial to perform quality control checks on the isolated nuclei to ensure their high quality for downstream applications such as next-generation sequencing (NGS). The value of isolated nuclei for molecular investigations has become apparent, and its use will only increase further as genomic and the multifaceted epigenomic mechanisms are being investigated (Figure 4D). With the development of novel cell-type-specific and epigenetic methodologies, nuclei have become a powerful tool for exploring different aspects of cellular specificity, especially in complex in-vivo systems or hard to dissociate tissue samples. Additionally, due to the simple acquisition, nuclei are gradually being incorporated into single-cell studies, providing an alternative to whole-cell data analyses. However, with the unique features that nuclei-based studies offer, it is crucial to better understand the challenges and limitations. Here, we presented the potential problems that nuclei-based studies still face, particularly concerning the transcriptomic analyses and data acquisition of nuclear RNA-seq compared to conventional RNA-seq approaches (Figure 4E). Further investigation should focus on the best potential strategy for the transcriptomic analyses of nuclear RNA. In terms of computational analysis, despite the lack of a concise pipeline or working strategy specialized for nucRNA-seq, the available tools for classic RNA-seq are shown to be adequate for nucRNA-seq data analysis. However, it is crucial to consider the appropriateness of the available tools and apply/omit them according to the biological question, experimental/sequencing strategy, and downstream data analysis, especially in combination with concomitant ‘omics’ approaches. Even though multiple studies have compared nuclear and cytoplasmic or total RNA fractions, particularly in association with single-cell/-nucleus RNA-seq, we display many open questions that remain to be addressed regarding the properties of nuclear RNA. For instance, future studies must determine the relevance of RIN values on nuclear RNA as well as the most suitable RNA library preparation strategy for nuclear RNA-seq. Understanding these differences between nuclear and whole-cell (or cytoplasmic) RNA will be crucial for developing and applying the next phase of nuclei-based studies at bulk- and single-cell levels. The application of long-read sequencing technologies such as Nanopore-seq and/or Pacbio could further uncover these differences, focusing mainly on differentially spliced transcripts as well as exon-intron occurrences. Being aware of these challenges can only improve and provide more accurate biological information for various scientific investigations. We are confident that with increasing interest and the use of nuclei-based studies, these challenges will be addressed, and as a result, the full potential of nuclei-based studies will be unlocked.

## Data Availability

Not applicable.
